# The effects of environmental tax reform on urban air pollution: A quasi-natural experiment based on the Environmental Protection Tax Law

**DOI:** 10.3389/fpubh.2022.967524

**Published:** 2022-08-12

**Authors:** Bingnan Guo, Yu Wang, Yu Feng, Chunyan Liang, Li Tang, Xiafei Yao, Feng Hu

**Affiliations:** ^1^School of Humanity and Social Science, Jiangsu University of Science and Technology, Zhenjiang, China; ^2^School of Economics, Shanghai University, Shanghai, China; ^3^Global Value Chain Research Center, Zhejiang Gongshang University, Hangzhou, China

**Keywords:** environmental tax reform, urban environment, air quality, difference-in-differences model, spatial spillover effect

## Abstract

Air pollution significantly impacts sustainable development and public health. Taking the implementation of China's Environmental Protection Tax Law in China as a quasi-natural experiment, this paper employs the difference-in-differences (DID) and spatial DID models to evaluate the effects of environmental tax reform on urban air pollution. The findings are as follows. (1) Environmental tax reform can significantly reduce urban air pollution, and a series of robustness tests have also been conducted to provide further evidence. (2) Green technology innovation and industrial structure upgrading from a vital transmission mechanism for environmental tax reform to improve air quality. (3) Environmental tax reform significantly inhibits urban air pollution in cities located north of the Qinling-Huaihe line and big cities. (4) Moreover, environmental tax reform not only promotes the improvement of local air quality but also has a significant negative spatial spillover effect, reducing air pollution in neighboring cities. The research conclusions provide theoretical support and policy suggestions for promoting sustainable economic development, rationally optimizing environmental protection tax policies and improving urban air quality.

## Introduction

Air pollution is the most severe environmental problem faced by countries worldwide, and how to improve air quality has been given high priority by governments. In recent decades, with the rapid development of urbanization and industrialization in China, energy consumption has increased rapidly, leading to increasingly severe air pollution in China (1). According to the 2021 China Eco-Environment Status Bulletin, of the 337 cities at the prefecture level and above in China, 40.1% still have air quality that seriously exceeds the standard. The 2020 Global Environmental Performance Index (EPI) shows that the total score of China is 37.3, and the EPI ranks 120th based on 180 countries and regions. These results severely impact sustainable economic development, public health, and government image. Against this background, the Chinese government has introduced many environmental policies and regulations to reduce environmental pollution. Accordingly, there is no doubt that it is of great theoretical value and policy significance to identify means of improving air quality.

Environmental regulation has been widely used in various countries and regions as an essential tool to alleviate environmental problems. However, due to the different selection of indicators and research samples, there is no unified conclusion on the relationship between environmental regulation and environmental pollution. Related studies have examined the pollution impact effects of different scales, periods, and types of environmental regulations (2–4). Some scholars believe environmental regulation can improve the ecological environment (5). Strict environmental regulation can encourage enterprises to conduct R&D and technological innovation (6), and improve the utilization rate and treatment rate of pollutants, thereby reducing environmental pollution (7). At the same time, environmental regulation can also inhibit the expansion of heavily polluting industries. The number of enterprises emitting large amounts of pollution is sharply reduced, indirectly leading to reducing environmental pollution (8). However, some studies have shown that environmental regulation cannot effectively reduce environmental pollution. Strict environmental regulations may reduce the economic efficiency of enterprises but are not conducive to improving environmental quality (9). Meanwhile, other studies have also concluded that there is uncertainty about the effect of environmental regulations on reducing environmental pollution (10).

As China's first single tax law to promote the construction of ecological civilization, the Environmental Protection Tax Law is an essential part of China's modern environmental governance system (11). It is of great significance to the management of environmental pollution. Before implementing the Environmental Protection Tax Law, China had long implemented the pollutant discharge fee system to replace the environmental protection tax system. The research on the pollution charge system is relatively affluent. The existing studies have verified that the pollutant discharge fee system plays a vital role in pollution control, energy conservation, and emission reduction (12, 13). However, some studies have also pointed out the shortcomings of the pollutant discharge fee system in the implementation process, such as low levy standards, many administrative interventions, non-standard levies, and lack of compulsory and standardized, which affect the effectiveness of its emission reduction (14). Theoretically, as a more compulsory, enforceable and supervisory environmental regulation tool, the environmental protection tax will bring cost pressure and supervisory pressure to force enterprises to undertake environmental treatment.

Environmental tax reform has important practical significance for promoting ecological civilization construction and improving urban air quality. Therefore, it is worth analyzing and discussing whether the Environmental Protection Tax Law implemented on January 1, 2018, can effectively protect and improve the environment and reduce pollutant emissions. This paper regards the implementation of the Environmental Protection Tax Law as a quasi-natural experiment. Based on the panel data of 283 cities in China from 2010 to 2019, the DID and PSM-DID methods are used to explore how the environmental tax reform can improve urban air quality and its transmission mechanism, heterogeneity and spatial spillover effects are empirically analyzed.

The main innovations of this research can be summarized as follows: First, in previous related studies, the measurement of environmental regulation often adopts a qualitative scoring method, single indicator method and comprehensive indicator method. These treatments do not effectively reflect the net effect of environmental regulation. In contrast, this paper adopts the difference-in-differences method, which can more accurately assess the pollution reduction effect of environmental tax reform and ensure the credibility of the estimation results. Second, most previous related studies were based on the pollutant charge system and the two-control zone policy before 2018. As China's first one-line tax law reflects the “green tax system,” there are still few studies on the Environmental Protection Tax Law. Based on this, this paper is the first to use the implementation of the Environmental Protection Tax Law as a policy to assess the effect of environmental tax reform on urban air pollution, enriching the literature on environmental governance. Third, based on the “Porter Hypothesis,” this paper analyzes that environmental tax reform can reduce urban air pollution by promoting green technology innovation and industrial structure upgrading from theoretical and empirical perspectives.

The rest of the paper is structured as follows. Section two presents the policy background and theoretical analysis. Section three introduces the research design, including model setting and data sources. Section four reports the main results. Section five constructs further analysis, including mechanism, heterogeneity, and spatial spillover effect tests. The last section is the conclusion and some policy recommendations.

## Policy background and research hypothesis

### Policy background for environmental protection tax law

Environmental protection tax was first proposed by Pigou, mainly through taxation to convert the external problems caused by environmental pollution into the internal costs of polluters (15). The environmental tax has become one of many countries' accepted macro-control measures (16). For example, the Netherlands pioneered a tax on surface water pollution in 1969; the U.S. Congress proposed a nationwide tax on sulfide emissions in 1971 and a tax on sulfur monoxide and nitric oxide emissions in 1987. The E.U. also has a comprehensive environmental protection tax system.

China's environmental tax system can be traced back to the late 1970s and early 1980s. The Environmental Protection Law (for Trial Implementation) promulgated in 1979 marked the initial establishment of the environmental tax system. In 1993, the Notice on the Collection of Sewage Discharge Fees was issued. Subsequently, in 2003, the State Council promulgated the Regulations on the Administration of the Collection and Use of Pollutant Discharge Fees, which clarified the budget management of pollutant discharge fees and the collection standards for wastewater and exhaust gas emissions (14). Although the pollutant discharge fee system has reduced pollutant emissions to some extent, China's environmental problems are still severe. Based on this, the National People's Congress promulgated the Environmental Protection Tax Law on December 15, 2016, which was officially implemented on January 1, 2018 (11). Meanwhile, the Environmental Protection Tax Law can solve the problems of insufficient law enforcement rigidity and administrative interference in the pollution discharge fee system, which is conducive to improving taxpayers' awareness of the ecological environment and strengthening the responsibility of enterprises for pollution control and emission reduction. In general, the Environmental Protection Tax Law provides a legal safeguard for environmental protection and represents a significant advance for China's environmental governance.

### Theoretical analysis and research hypothesis

Environmental regulation is essentially based on the negative externality of pollution. It regulates the activities of various social agents, including enterprises, by formulating corresponding systems and implementing them to achieve the primary goal of environmental protection. Generally speaking, the impact of environmental regulation on environmental quality is mainly transmitted from three dimensions: source, process and end-of-pipe treatment. The environmental protection tax mainly focuses on end-of-pipe treatment, targeting the pollutants already produced to effectively treat them and minimize the total pollutants (17). Environmental protection tax is a specific behavior tax levied by enterprises, producers, and operators that directly discharge taxable pollutants into the environment. Taxable pollutants include air pollutants, water pollutants, solid waste and noise. Enterprises are the main body of pollution discharge and the critical link of environmental governance. The environmental tax reform has increased the cost of enterprises' pollution discharge, which has prompted enterprises to reduce pollution discharge and use more renewable energy in the production process. At the same time, after implementing the Environmental Protection Tax Law, the environmental protection tax all belong to the fiscal revenue of the local government. This makes local governments have a greater willingness and ability to invest in pollution monitoring, thereby improving environmental quality. Based on this, this paper proposes the first hypothesis:

Hypothesis 1: Environmental tax reform can reduce urban air pollution.

As a market-incentivized environmental regulation, the environmental protection tax plays a vital role in green technology innovation. On the one hand, the Environmental Protection Tax Law has raised the levy standards for pollutant emissions, which has brought higher pressure on enterprises to reduce emission reduction costs. According to the theory of enterprise competitiveness, external pressure can help enterprises overcome inertia and stimulate innovative thinking, and promote enterprises to carry out green technology innovation (18). On the other hand, environmental regulation reduces uncertainty about the value of corporate investments in the environmental sector and can affect corporate expectations. The Environmental Protection Tax Law implementation shows the government's determination to protect the environment and the direction of policy development. Therefore, enterprises will carry out green technology innovation for long-term interests (19). At the same time, with the improvement of green technology innovation, enterprises can improve resource utilization efficiency and produce clean and non-polluting products, thereby reducing pollution emissions in the production process (20). Based on this, this paper proposes the first hypothesis:

Hypothesis 2: Environmental tax reform can improve urban air quality by promoting green technology innovation.

The environmental protection tax is not to make enterprises pay more taxes, but according to the tax system design of “more emissions, more payments, fewer emissions, fewer payments, no payments, no payments” and to subsidize enterprises that reduce the concentration of emissions, to improve the innovative power of enterprises. This will optimize low-end industries with high pollution, high energy consumption and high emissions to high-end industries with zero pollution, low energy consumption and zero emissions, and promote the development of strategic emerging industries and high-end service industries, thereby realizing the upgrading of the industrial structure (21). With the upgrading of the industrial structure, new industries will use more non-polluting and clean production factors for production. The sulfur dioxide, smoke, and dust emission in the industrial production process will be reduced (22). Based on this, this paper proposes the first hypothesis:

Hypothesis 3: Environmental tax reform can reduce urban air pollution by promoting industrial structure upgrading.

## Research design

### Model setting

#### DID model

The DID method can efficiently identify the causal effect of the external policy shocks by comparing the net effect between the treatment and control groups. In order to protect the environment and reduce pollutant emissions, the Environmental Protection Tax Law came into effect on January 1, 2018. Hence, taking the Environmental Protection Tax Law as a quasi-natural experiment, this paper applies the DID method to examine environmental tax reform's effects on urban air pollution. The specific model is as follows (23):


(1) $$\begin{array}{c} pollution_{it}=\alpha_{0}+\alpha_{1}did_{it}+\alpha_{c}X_{it}+\gamma_{t}+\mu_{i}+\varepsilon_{it} \end{array}$$

(2) $$\begin{array}{c} where\;did_{it}=group_{i}\;\times \;time_{t} \end{array}$$

In this formula, *i* represents the city, *t* represents the year. *pollution*_*it*_ represents the air pollution of the city *i* in the year *t*. Urban air pollution includes two indicators in this study: industrial sulfur dioxide emissions per capita (*lnso*_2_) and industrial smoke and dust emissions per capita (*lnsmoke*). *group*_*it*_ presents city dummies; its value is 1 if city *i* raises the standard of environmental protection tax, and 0 otherwise^1^. *time*_*it*_ presents time dummy variable; its value is 1 if the year is greater than or equal to 2018, and 0 otherwise. *X*_*it*_ are the control variables affecting the urban air pollution for city *i* at year *t*. γ_*t*_is the year fixed effect. μ_*i*_is the city fixed effect. ε_*it*_ is the random error term. At the same time, the robust standard errors are clustered to the city level.

The control variables of this study are as follows: The level of economic development (*lnpgdp*) is expressed in terms of regional GDP per capita. Government regulation (*gov*) is expressed as the ratio of local fiscal expenditure to GDP. Population density (*lndensity*) is expressed as the ratio of the total population at the end of the year to the land area of the administrative district. The green coverage rate (*greenratio*) is expressed by the ratio of the green area of the built-up area to the built-up area. The foreign direct investment (*fdi*) is expressed by the actual foreign direct investment ratio to the GDP.

#### Mechanism test model

A two-stage mechanism analysis model is adopted to analyze the influence mechanism of environmental tax reform on urban air pollution (24). In the first stage, the effects of environmental tax reform on green technology innovation and industrial structure upgrading are examined using Eq. (3). In the second stage, the effects of green technology innovation and industrial structure upgrading on urban air pollution are checked using Eq. (4). The model Settings are as follows:


(3) $$\begin{array}{c} mech_{it}=\beta_{0}+\beta_{1}did_{it}+\beta_{c}X_{it}+\gamma_{t}+\mu_{i}+\varepsilon_{it} \end{array}$$

(4) $$\begin{array}{c} pollution_{it}=\vartheta_{0}+\vartheta_{1}mech_{it}+\vartheta_{c}X_{it}+\gamma_{t}+\mu_{i}+\varepsilon_{it} \end{array}$$

Where *mech*_*it*_is the mediator variable, including green technology innovation and industrial structure upgrading. Green technology innovation (*gtp*) is measured by the number of green patent applications per 10,000 people (25). Industrial structure upgrading (*is*) is the added value ratio between the tertiary industry and the second industry (26). The meaning of other variables is the same as formula (1).

#### Spatial DID model

The implicit assumption of the traditional difference-in-differences model is that any individual will not be affected by whether other individuals are treated or not, so the neglect of spatial correlation between cities will lead to biased estimation results. Hence, it is necessary to employ an SDID model to study the spatial spillover effect of environmental tax reform on urban air pollution (27). The model is set as follows:


(5) $$\begin{array}{r} \begin{array}{c} pollution_{it}=\pi_{0}+\rho Wpollution_{it}+\pi_{1}did_{it}+\theta Wdid_{it}\\ +\pi_{c}X_{it}+\delta WX_{it}+\gamma_{t}+\mu_{i}+\varepsilon_{it} \end{array} \end{array}$$

In Eq. (5), *Wpollution*_*it*_ is the spatial lag in urban air pollution, *Wdid*_*it*_ is the spatial lag of environmental tax reform, ρ is the spatial autocorrelation coefficient of urban air pollution, π_1_ is the coefficient of the effect of environmental tax reform on local air pollution, θ is the coefficient of the impact of environmental tax reform on air pollution in neighboring cities, and *W* is a 283^*^283 geographic distance spatial weight matrix. The meaning of other variables is the same as formula (1).

### Data sources

Our study sample contains 283 prefecture-level and above cities in China from 2010 to 2019, and these city-level data are driven from China City Statistics Yearbook, China Environmental Statistics Yearbook, and the EPS database. Meanwhile, the number of green patent applications is based on the International Patent Classification (IPC) green list code issued by the World Intellectual Property Organization (WIPO), and is collated according to the patent application information provided by the State Intellectual Property Office of China. The descriptive statistics for the main variables are presented in Table 1.

**Table 1:** Descriptive statistics for the variables.

**Variables**	**Observations**	**Mean**	**Standard deviation**	**Minimum**	**Maximum**
*lnso_2_*	2830	−5.027	1.277	−12.59	−1.229
*lnsmoke*	2830	−8.913	1.297	−13.55	−3.669
*did*	2830	0.086	0.280	0	1
*gtp*	2830	0.689	1.492	0.003	22.84
*is*	2830	1.101	0.682	0.011	6.533
*lnpgdp*	2830	10.65	0.594	8.576	13.06
*gov*	2830	0.205	0.186	0.029	3.512
*lndensity*	2830	5.748	0.917	1.619	7.923
*greenratio*	2830	0.474	0.470	0.003	11.39
*fdi*	2830	0.020	0.055	0.001	1.371

## Empirical results and analysis

### Common trend test

The premise of practical estimation of the DID method is to satisfy the parallel trend hypothesis. In other words, if the Environmental Protection Tax Law is not implemented, the variation trend of urban air pollution in the treatment and control groups should be the same. Furthermore, the benchmark regression results reflect the average impact of the environmental tax reform on urban air pollution rather than differences in effect over time. Consequently, this paper uses the event analysis method to construct the following model (28):


$$\begin{array}{c} \begin{array}{c} pollution_{it}\;=\;\alpha_{0}+\sum_{k=-7,k\neq -1}^{1}\alpha_{k}did_{it}^{k}+\alpha_{c}X_{it}+\gamma_{t}\\ +\mu_{i}+\varepsilon_{it}\; \end{array} \end{array}$$

Where $did_{it}^{k}$ is a dummy variable. Provided that the year when city *i* is affected by the Environmental Protection Tax Law is *s* (*s* = 2018), then we set *t*−*s* = *k*. When *k* is negative, if *t* is smaller than the policy implementation time, then we set $did_{it}^{k}=1$; otherwise, we set $did_{it}^{k}=0$. When *k* is no smaller than 0, if *t* is larger than the policy implementation time, then we set $did_{it}^{k}=1$; otherwise, we set $did_{it}^{k}=\;0$.

**Figure 1:**
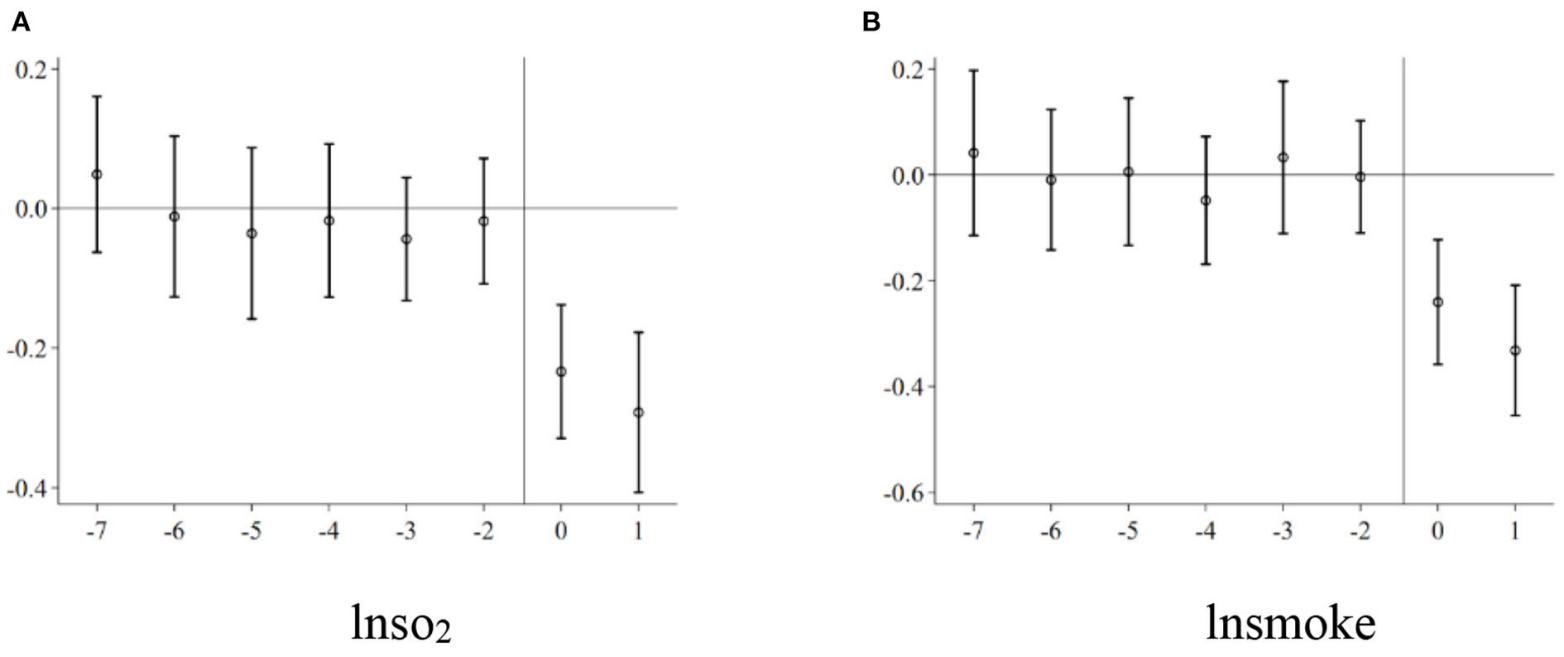
Common trend test. **(A)** lnso_2_. **(B)** lnsmoke.

Figure 1 shows the estimated coefficients of α_*k*_ under the 90% confidence intervals. Figure 1A shows the impact of environmental tax reform on industrial sulfur dioxide emissions per capita, and Figure 1B shows the impact of environmental tax reform on industrial smoke and dust emissions per capita. It can be seen that the estimated coefficients of α_*k*_ are insignificant before implementing the Environmental Protection Tax Law, which means that there is no significant difference in urban air pollution between the treatment and control groups before policy implementation. At the same time, after the implementation of the Environmental Protection Tax Law, the industrial sulfur dioxide emissions per capita and industrial smoke and dust emissions per capita have been significantly reduced. Therefore, the parallel trend hypothesis was verified.

### Main results

The net effect of environmental tax reform on urban air pollution is evaluated, and the empirical results are reported in Table 2, in which the industrial sulfur dioxide emissions per capita (*lnso*_2_) and industrial smoke and dust emissions per capita (*lnsmoke*). The estimated coefficient of the interaction term captures the average effect. In each regression, the coefficient of the interaction term is significantly negative at the 1% level.

**Table 2:** Effects of environmental tax reform on urban air pollution.

	**DID**		**PSM-DID**	
	***lnso_2_***	***lnsmoke***	***lnso_2_***	***lnsmoke***
	**(1)**	**(2)**	**(3)**	**(4)**
*did*	−0.253***	−0.288***	−0.256***	−0.303***
	(0.073)	(0.075)	(0.075)	(0.076)
Control variables	Yes	Yes	Yes	Yes
City FE	Yes	Yes	Yes	Yes
Year FE	Yes	Yes	Yes	Yes
Observations	2830	2830	2770	2770
R-squared	0.891	0.840	0.888	0.841

(1) The values in parentheses are robust standard errors for clustering to the city level; (2) ^***^, ^**^, ^*^ represent statistical significance at the 1, 5, and 10% levels, respectively.

The benchmark results for the DID model are presented in the first two columns of Table 2. The results show that the coefficients of industrial sulfur dioxide emissions per capita and industrial smoke and dust emissions per capita are all significantly negative. This preliminarily confirms that environmental tax reform can significantly reduce air pollution and improve air quality in cities.

Meanwhile, a potential concern with DID method is that the treatment and control groups may differ in ways that would affect their trends over time, or their compositions may change over time (29). Hence, this paper uses the difference-in-differences propensity score matching (PSM-DID) method to select suitable samples for further comparison and provides unbiased estimation results by effective matching (30). Specifically, we take the urban air pollution as the outcome variables and the control variables in Eq. 1 as covariates and carry out corresponding matching according to the one-to-one neighbor matching method with put-back. Columns (3) and (4) in Table 2 show the regression results for the PSM-DID method. It can be seen that the coefficients are both negative at the 1% level, indicating that environmental tax reform has a noticeable lowing effect on urban air pollution.

Taken together, environmental tax reform can significantly improve urban air quality by reducing industrial sulfur dioxide emissions per capita and industrial smoke and dust emissions per capita.

### Removing samples that are potentially affected by other policies

During the study period, the Chinese government has also implemented a series of policy tools to reduce environmental pollution, which may lead to overestimating the impact of environmental tax reform on urban air pollution. This paper controls the interference of other policies on the results to solve this problem. It is argued that environmental pollution is affected by the new energy demonstration program and low-carbon city pilot policy (30, 31). On the one hand, the new energy demonstration program could reduce environmental pollution through technological innovation and resource allocation (32). On the other hand, the low-carbon city pilot policy has an important impact on promoting green technology innovation and reducing carbon emissions. Therefore, this paper deletes the cities that implement the new energy demonstration program and the low-carbon city pilot policy in the benchmark regression model to exclude these policies' impact. The regression results are shown in Table 3. When the study samples that are potentially affected by other policies are removed, it can be seen that the regression coefficient remains significantly negative.

**Table 3:** Effects of policy uniqueness test.

	**Pollution levy standard system**		**Low carbon city pilot policy**	
	***lnso_2_***	***lnsmoke***	***lnso_2_***	***lnsmoke***
	**(1)**	**(2)**	**(3)**	**(4)**
*did*	−0.177***	−0.199***	−0.452***	−0.340**
	(0.046)	(0.046)	(0.133)	(0.158)
Control variables	Yes	Yes	Yes	Yes
City FE	Yes	Yes	Yes	Yes
Year FE	Yes	Yes	Yes	Yes
Observations	1132	1132	1620	1620
R-squared	0.940	0.947	0.881	0.844

(1) The values in parentheses are robust standard errors for clustering to the city level; (2) ^***^, ^**^, ^*^ represent statistical significance at the 1, 5, and 10% levels, respectively.

### Other robustness checks

#### Placebo test

**Figure 2:**
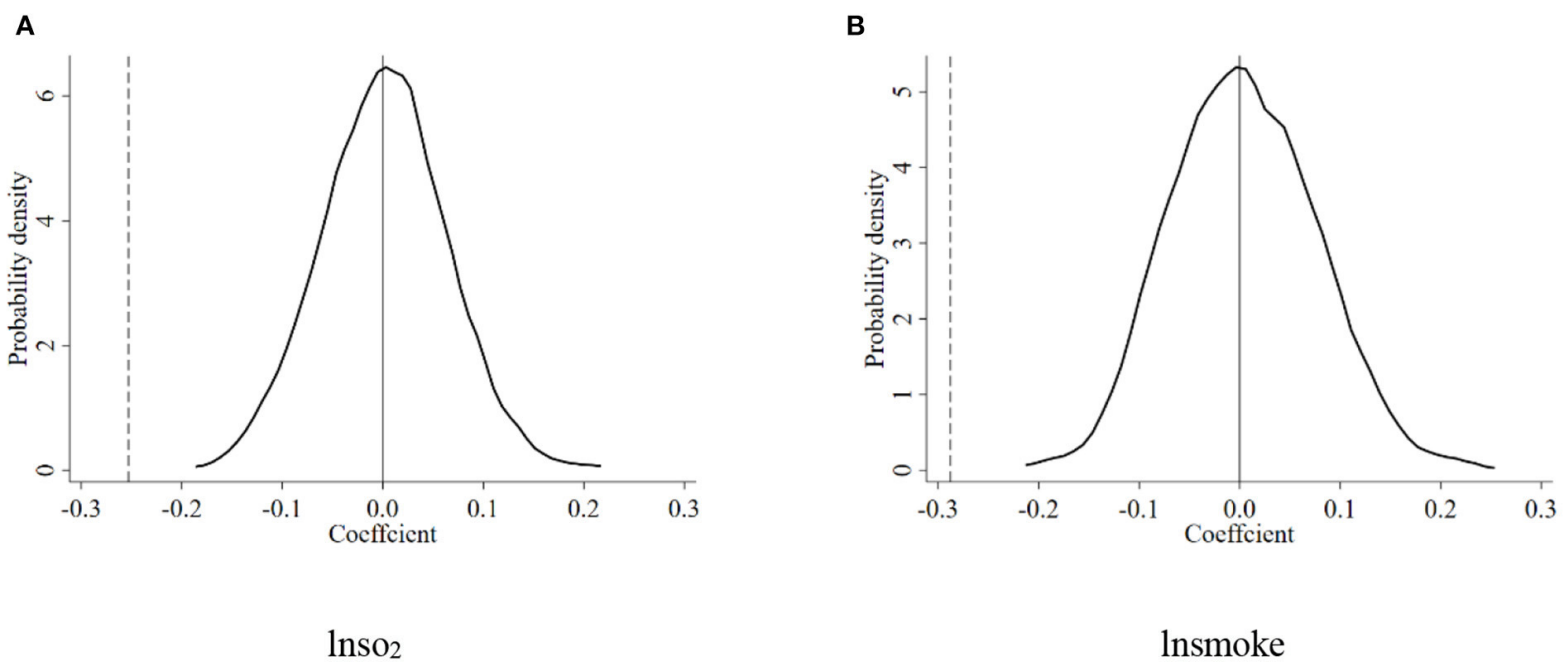
Results of the kernel density distribution of the DID of placebo test. **(A)** lnso_2_. **(B)** lnsmoke.

Another concern about the DID method is other non-observed and omitted variables. Therefore, this paper randomly selected 121 samples as the treatment group from the total sample and a year as the policy implementation time during the study period for counterfactual testing (28). Then, we repeated 1000 estimates based on the benchmark regression results in columns (1) and (2) of Table 2. The probability density distribution of the placebo test regression coefficients is shown in Figure 2. The estimated coefficients are centered around 0, while the benchmark regression result is outside the entire distribution. Hence, it meets the expectations of the Placebo test.

#### Replace the explained variable

The haze pollution caused by fine particulate matter (PM 2.5) emissions has drawn extensive attention. Therefore, this paper selects PM2.5 as a proxy variable for urban air pollution (33), then re-estimates the impact of environmental tax reform on urban air pollution. The PM 2.5 data were released by NASA Socioeconomic Data and Applications Center (34). Furthermore, we use ArcGIS to parse it into the city's annual average concentration data. The regression results are shown in columns (1) and (2) of Table 4. It can be seen that the regression coefficients are significantly negative regardless of whether control variables are added, which also proves the robustness of the benchmark regression results.

**Table 4:** The results of the robustness test.

	**Replace the explained variable**		**Delete center city**		**Province clustering**	
	***PM 2.5***	***PM 2.5***	***lnso_2_***	***lnsmoke***	***lnso_2_***	***lnsmoke***
	**(1)**	**(2)**	**(3)**	**(4)**	**(5)**	**(6)**
*did*	−6.829***	−6.331***	−0.248***	−0.265***	−0.253**	−0.288**
	(1.641)	(1.523)	(0.080)	(0.081)	(0.118)	(0.136)
Control variables	No	Yes	Yes	Yes	Yes	Yes
City FE	Yes	Yes	Yes	Yes	Yes	Yes
Year FE	Yes	Yes	Yes	Yes	Yes	Yes
Observations	2480	2480	2830	2830	2830	2830
R-squared	0.887	0.841	0.839	0.840	0.891	0.840

(1) The values in parentheses are robust standard errors for clustering to the city level; (2) ^***^, ^**^, ^*^ represent statistical significance at the 1, 5, and 10% levels, respectively.

#### Delete center city

The administrative power of the government in different-level cities may be quite different, and higher-level cities have far more resources than ordinary prefecture-level cities (30). Therefore, this paper deletes the provincial capital cities, sub-provincial cities and municipalities directly in the study sample and only retains the samples of ordinary prefecture-level cities for regression. The results are shown in columns (3) and (4) of Table 4. It can be seen that the environmental tax reform still significantly reduces urban air pollution, further verifying the robustness of the benchmark regression results.

#### Province clustering

In the above empirical analysis, this paper clusters the robust standard errors to the city level, but different cities in the same province may be affected by policies at the provincial level. At the same time, there is greater independence between provinces. The higher the level of clustering, the weaker the underlying assumptions. Therefore, to ensure the regression results' robustness, this paper clusters the robust standard errors to the provincial level. The results are shown in columns (5) and (6) of Table 4, and it can be seen that the regression results are still significantly negative.

## Further analysis

### Impact mechanism test results and discussion

The benchmark regression results demonstrate that the environmental tax reform contributes to the improvement of urban air quality according to reducing industrial sulfur dioxide emissions per capita and industrial smoke and dust emissions per capita. Therefore, to further analyze the influence mechanism of environmental tax reform on urban air pollution, green technology innovation and industrial structure upgrading are selected as the mechanism variables in this paper to verify hypotheses 2 and 3. The regression results are shown in Table 5. Specifically, in columns (1) and (4), the coefficients are significantly positive, indicating that environmental tax reform can promote green technology innovation and industrial structure upgrading. The results in columns (2), (3), (5) and (6) show that green technology innovation and industrial structure upgrading effectively reduce urban air pollution. Therefore, the improvement of environmental tax reform on urban air quality by promoting green technology innovation and industrial structure upgrading.

**Table 5:** Analysis results of influence mechanism.

	**Green technology innovation**			**Industrial structure upgrading**		
	***gtp***	***lnso_2_***	***lnsmoke***	***is***	***lnso_2_***	***lnsmoke***
	**(1)**	**(2)**	**(3)**	**(4)**	**(5)**	**(6)**
*did*	0.170***			0.080*		
	(0.059)			(0.042)		
*gtp*		−0.389***	−0.159***			
		(0.046)	(0.037)			
*is*					−0.715***	−0.380***
					(0.081)	(0.070)
Control variables	No	Yes	Yes	Yes	Yes	Yes
City FE	Yes	Yes	Yes	Yes	Yes	Yes
Year FE	Yes	Yes	Yes	Yes	Yes	Yes
Observations	2480	2480	2830	2830	2830	2830
R-squared	0.872	0.745	0.730	0.827	0.755	0.735

(1) The values in parentheses are robust standard errors for clustering to the city level; (2) ^***^, ^**^, ^*^ represent statistical significance at the 1, 5, and 10% levels, respectively.

On the one hand, the environmental tax reform brings higher cost pressure for enterprises to reduce emissions and shows the government's determination to control environmental pollution. These external pressures will encourage enterprises to carry out green technology innovation, thereby reducing or even avoiding the additional costs of environmental taxes (35). On the other hand, the reasonable implementation of environmental regulations can optimize and upgrade local low-end industries with high pollution, high energy consumption, and high emissions to high-end industries with zero pollution, low energy consumption, and low emissions. This not only cultivates strategic emerging industries and high-end service industries but also improves urban air quality (36). Therefore, hypothesis 2 and hypothesis 3 of this paper are verified.

### Heterogeneity analysis

#### Impact of city location on environmental tax reform

The Qinling-Huaihe River line is the geographical boundary between the north and the south of China and, to a certain extent, the boundary of central heating in winter. Heating cities in northern China consume many fossil fuels in winter, which significantly impacts air pollution (37). Hence, this paper examines the impact of environmental tax reform on urban air pollution by dividing cities into two types: north of the Qinling-Huaihe line and south of the Qinling-Huaihe line^2^. Columns (1) to (4) in Table 6 show the results of each sub-sample. The results show that environmental tax reform significantly reduces urban air pollution in the north of the Qinling-Huaihe line. Meanwhile, the impact coefficients on the south of the Qinling-Huaihe line are negative but insignificant. The results indicate that north of the Qinling-Huaihe line performs better in environmental tax reform than south of the Qinling-Huaihe line. The main reason may be that the industrial structure level of northern cities is relatively low, mainly the secondary industry with high pollution and high energy consumption. At the same time, a large number of fossil fuels are consumed for heating in winter, which leads to severe air pollution. Therefore, northern cities' environmental protection tax reform may have more substantial marginal effects.

**Table 6:** Comparison of environmental tax reform effects in different regions.

	**North of the Qinling-Huaihe line**		**South of the Qinling-Huaihe line**	
	***lnso_2_***	***lnsmoke***	***lnso_2_***	***lnsmoke***
	**(1)**	**(2)**	**(3)**	**(4)**
*did*	−0.517***	−0.533***	−0.063	−0.110
	(0.098)	(0.117)	(0.103)	(0.094)
Control variables	Yes	Yes	Yes	Yes
City FE	Yes	Yes	Yes	Yes
Year FE	Yes	Yes	Yes	Yes
Observations	1080	1080	1530	1530
R-squared	0.908	0.865	0.869	0.821

(1) The values in parentheses are robust standard errors for clustering to the city level; (2) ^***^, ^**^, ^*^ represent statistical significance at the 1, 5, and 10% levels, respectively.

#### Impact of city size on environmental tax reform

City size also has a significant impact on air pollution. On the one hand, big cities have an economic agglomeration effect, attracting high-end talents, capital, and technology. Therefore, big cities can better solve environmental pollution by optimizing resource allocation (38). On the other hand, there is a crowding effect in big cities. Big cities have a stronger demand for energy consumption, which leads to the deterioration of the ecological environment (39). Therefore, to further investigate the impact of the city size on the effect of environmental tax reform, this paper divides the sample cities into “big cities” and “small cities.” The classification of city size is mainly based on the “Notice on Adjusting the Criteria for Urban Size Division” issued by the State Council in 2014. In our study, cities with a permanent population of more than 3 million are regarded as big cities, and those with less than 3 million are regarded as small cities. The results are shown in Table 7.

**Table 7:** Heterogeneity analysis of city size.

	**Big cities**		**Small cities**	
	***lnso_2_***	***lnsmoke***	***lnso_2_***	***lnsmoke***
	**(1)**	**(2)**	**(3)**	**(4)**
*did*	−0.218**	−0.272***	−0.237	−0.282
	(0.085)	(0.095)	(0.149)	(0.254)
Control variables	Yes	Yes	Yes	Yes
City FE	Yes	Yes	Yes	Yes
Year FE	Yes	Yes	Yes	Yes
Observations	1560	1560	1270	1270
R-squared	0.893	0.828	0.889	0.841

(1) The values in parentheses are robust standard errors for clustering to the city level; (2) ^***^, ^**^, ^*^ represent statistical significance at the 1, 5, and 10% levels, respectively.

For the big cities, environmental tax reform still presents a significant negative correlation with lnso_2_ and lnsmoke. However, for the small cities, the coefficients are insignificant. One possible explanation is that those big cities have better resource endowments and economic development conditions and shoulder relatively heavy social governance responsibilities and environmental protection responsibilities. Therefore, these cities actively improve air quality, promoting high-quality economic development.

### Test of the spatial spillover effect

The premise of using the SDID model is to satisfy the spatial correlation. This paper calculates the global Moran's I index of urban air pollution from 2010 to 2019. Table 8 shows the regression results. The results show that the Moran's I index of urban air pollution is significantly positive at the 1% level, confirming that air pollution among different cities has significant positive spatial dependence. Meanwhile, these results also demonstrate that it is rational to use an SDID model. Hence, this paper adopts a two-way fixed effects model to evaluate the spillover effects of environmental tax reform.

**Table 8:** Moran's I index of lnso_2_ and lnsmoke.

	***lnso***_**2**_		***lnsmoke***	
	**Moran's I**	***Z*** **-value**	**Moran's I**	***Z*** **-value**
2010	0.192***	8.278	0.184***	7.990
2011	0.221***	9.506	0.183***	7.938
2012	0.208***	8.977	0.200***	8.714
2013	0.216***	9.316	0.228***	9.931
2014	0.197***	8.473	0.227***	9.913
2015	0.199***	8.593	0.245***	10.519
2016	0.180***	7.770	0.230***	9.880
2017	0.145***	6.298	0.202***	8.708
2018	0.109***	4.745	0.217***	9.350
2019	0.117***	5.099	0.221***	9.511

^***^, ^**^, ^*^ represent statistical significance at the 1, 5, and 10% levels, respectively.

The spatial model differs from the traditional econometric model in that the estimated coefficients of its regression results cannot directly reflect the marginal effects of the explained variables. Therefore, this paper decomposed the regression results to obtain the direct, indirect, and total effects. The regression results are shown in Table 9. The results demonstrate that the direct effect of environmental tax reform on urban air pollution is significantly negative, which further confirms the emissions reduction effect of environmental tax reform. Meanwhile, the results of the indirect effect are also significantly negative, meaning that environmental tax reform has a positive spillover effect on regional air quality. On the one hand, air pollution has a spillover effect, so the pollution reduction effect brought about by the environmental tax reform in neighboring areas may spread to the local area, thereby effectively improving the local air quality. On the other hand, local governments will compete to improve the level of environmental regulation due to NIMBYism and their pursuit of liquidity factors that prefer a high-quality environment (40).

**Table 9:** The results of the spatial difference-in-differences model.

	***lnso***_**2**_		***lnsmoke***	
	**(1)**	**(2)**	**(3)**	**(4)**
Direct effect	−0.135*	−0.105***	−0.052***	−0.083***
	(0.074)	(0.036)	(0.016)	(0.023)
Indirect effect	−1.820***	−1.091***	−1.302***	−1.130***
	(0.300)	(0.252)	(0.198)	(0.228)
Total effect	−1.955***	−1.196***	−1.354***	−1.213***
	(0.275)	(0.228)	(0.159)	(0.192)
Control variables	No	Yes	No	Yes
City FE	Yes	Yes	Yes	Yes
Year FE	Yes	Yes	Yes	Yes
*Spa-rho*	0.854***	0.790***	0.702***	0.695***
	(0.015)	(0.019)	(0.023)	(0.024)
*Sigma2*	0.187***	0.178***	0.274***	0.272***
	(0.005)	(0.004)	(0.007)	(0.007)
Observations	2830	2830	2830	2830
R-squared	0.172	0.250	0.040	0.056

(1) The values in parentheses are robust standard errors for clustering to the city level; (2) ^***^, ^**^, ^*^ represent statistical significance at the 1, 5, and 10% levels, respectively.

## Conclusion and policy implications

Based on the panel data of 283 cities in China from 2010 to 2019, this paper regards implementing the environmental protection tax law as a quasi-natural experiment to empirically test the impact of environmental tax reform on urban air pollution. The findings show that: (1) Environmental tax reform has significantly reduced urban air pollution. This conclusion still holds after a series of robustness tests such as PSM-DID, parallel trend test, and placebo test. (2) The heterogeneity study shows that the environmental tax reform has a more substantial reduction effect on air pollution in cities north of the Qinling-Huaihe line than in cities south of the line; meanwhile, the environmental tax reform has a more significant impact effect on big cities. (3) The mechanism test shows that environmental tax reform improves urban air quality by promoting green technology innovation and industrial structure upgrading. (4) Environmental tax reform not only improves local air quality but also has a reduced effect on air pollution in neighboring cities.

Our study adds to a growing body of research exploring the environmental tax reform and provides more definitive evidence from the perspective of prefecture-level cities. This paper proposes policy recommendations based on the findings above. First, actively strengthen the implementation of the Environmental Protection Tax Law and explore more reasonable environmental tax regulations. Collecting environmental tax requires cooperation between the tax department and the environmental protection department. An information-sharing mechanism between the two departments should be established to improve the efficiency of tax collection and management, thereby promoting the improvement of urban air quality. Second, promote green technology innovation and industrial structure upgrading. Promote urban air quality improvement through cleaner production technologies and the development of high-end industries. Third, in the case of significant differences in the endowment conditions of each city, relevant policies should be formulated according to local conditions.

There are still some limitations to be considered to study further. On the one hand, environmental protection taxes have an impact on various pollutants, and this paper only studies air pollution due to data limitations. On the other hand, our results are more applicable to the Chinese city. However, as the micro-subject of pollution emissions, the research on the impact of an environmental protection tax on corporate pollution is also worthy of attention. We believe that further work will show a useful supplement in these aspects.

## Data availability statement

The original contributions presented in the study are included in the article/supplementary material, further inquiries can be directed to the corresponding author/s.

## Author contributions

BG and YW designed the study, performed the research, analyzed data, and wrote the paper. YF and CL collected most of the data. LT and XY checked the spelling of the paper and corrected the mistakes. FH provided fund support and suggestions on revising the paper. All authors contributed to the article and approved the submitted version.

## Funding

This work was supported the Postgraduate Research and Practice Innovation Program of Jiangsu Province (Grant Number KYCX21_3411).

## Conflict of interest

The authors declare that the research was conducted in the absence of any commercial or financial relationships that could be construed as a potential conflict of interest.

## Publisher's note

All claims expressed in this article are solely those of the authors and do not necessarily represent those of their affiliated organizations, or those of the publisher, the editors and the reviewers. Any product that may be evaluated in this article, or claim that may be made by its manufacturer, is not guaranteed or endorsed by the publisher.

## Footnotes

^1^During the implementation of the Environmental Protection Tax Law, each province can independently determine the tax rate according to the local environmental conditions. Some provinces use the original pollution discharge fee collection standard as the environmental protection tax standard, while Hebei, Henan, Jiangsu, Shandong, Hunan, Sichuan, Chongqing, Guizhou, Hainan, Guangxi, Shanxi and Beijing have raised tax rates.

^2^Cities south of the Qinling-Huai River line do not have central heating, while some cities north of the line—Heilongjiang, Jilin, Liaoning, Beijing, Tianjin, Hebei, Shanxi, Inner Mongolia, Shandong, Gansu, Qinghai, Ningxia, Xinjiang, Tibet, Jiangsu Xuzhou, Henan and Shaanxi—use collective heating.
